# The Susceptibilities of Respiratory Syncytial Virus to Nucleolin Receptor Blocking and Antibody Neutralization Are Dependent upon the Method of Virus Purification

**DOI:** 10.3390/v9080207

**Published:** 2017-08-03

**Authors:** Leanne M. Bilawchuk, Cameron D. Griffiths, Lionel D. Jensen, Farah Elawar, David J. Marchant

**Affiliations:** Department of Medical Microbiology and Immunology, Li Ka Shing Institute of Virology, University of Alberta, Edmonton, AB T6G 2R3, Canada; lbilawch@ualberta.ca (L.M.B.); cdg1@ualberta.ca (C.D.G.); ldjensen@ualberta.ca (L.D.J.); elawar@ualberta.ca (F.E.)

**Keywords:** respiratory syncytial virus, fast protein liquid chromatography, purification, sucrose gradient ultracentrifugation, density, mass spectrometry, transmission electron microscopy, antibody neutralization, nucleolin

## Abstract

Respiratory Syncytial Virus (RSV) that is propagated in cell culture is purified from cellular contaminants that can confound experimental results. A number of different purification methods have been described, including methods that utilize fast protein liquid chromatography (FPLC) and gradient ultracentrifugation. Thus, the constituents and experimental responses of RSV stocks purified by ultracentrifugation in sucrose and by FPLC were analyzed and compared by infectivity assay, Coomassie stain, Western blot, mass spectrometry, immuno-transmission electron microscopy (TEM), and ImageStream flow cytometry. The FPLC-purified RSV had more albumin contamination, but there was less evidence of host-derived exosomes when compared to ultracentrifugation-purified RSV as detected by Western blot and mass spectrometry for the exosome markers superoxide dismutase [Cu-Zn] (SOD1) and the tetraspanin CD63. Although the purified virus stocks were equally susceptible to nucleolin-receptor blocking by the DNA aptamer AS1411, the FPLC-purified RSV was significantly less susceptible to anti-RSV polyclonal antibody neutralization; there was 69% inhibition (*p* = 0.02) of the sucrose ultracentrifugation-purified RSV, 38% inhibition (*p* = 0.03) of the unpurified RSV, but statistically ineffective neutralization in the FPLC-purified RSV (22% inhibition; *p* = 0.30). The amount of RSV neutralization of the purified RSV stocks was correlated with anti-RSV antibody occupancy on RSV particles observed by immuno-TEM. RSV purified by different methods alters the stock composition and morphological characteristics of virions that can lead to different experimental responses.

## 1. Introduction

Human Respiratory Syncytial Virus (RSV) is an enveloped, single-stranded, negative-polarity RNA virus of the family *Pneumoviridae* that poses a massive burden on worldwide health [1,2]. In the USA alone, over 2 million RSV-infected infants require medical attention annually, making it one of the most common causes of infant hospital admissions in North America [1,3]. Despite this burden of disease, there are few licensed therapeutics available to treat RSV infections, and vaccine development has long been hindered due to adverse outcomes, including two infant deaths in a clinical trial that was conducted in 1967 [4].

There has been a recent surge in RSV vaccine and therapeutic development (reviewed in [5]), however, determining the efficacy of new vaccines and drugs entails extensive in vitro and in vivo testing that requires purified virus stocks. The purification of a virus serves two purposes: to concentrate virus particles and to remove the bulk of cell-derived matter. Cell-derived proteins that contaminate unpurified virus stocks typically include growth factors, cytoskeleton proteins, and chemokines and cytokines that potentially confound experimental analyses [6,7,8]. However, the term ‘purify’ is a misnomer because it is impossible to remove all traces of cell-derived factors from virus stocks [9]. Therefore, a careful analysis of RSV stocks for experimental vaccine and therapeutic validation is required to verify the quality of the stocks and provide a precedent for standardization.

A particular challenge in purifying, storing, and manipulating RSV is its apparent lability [10,11,12,13], making it a difficult virus with which to work. Storage at −90 °C for longer than 3 weeks, or a single freeze–thaw cycle, significantly reduces the infectivity of RSV stocks [11]. As such, different buffers have been tested as a medium for the purification of RSV by density gradient centrifugation, including iodixanol and sucrose [14,15]. Sucrose acts as a cryopreservative of RSV infectivity [12] that is an added benefit to purifying RSV by sucrose gradient ultracentrifugation. However, it is not known whether iodixanol can help preserve RSV, and the iodixanol and sucrose methods of RSV purification have not been compared directly.

Although density gradient centrifugation is a proven and popular method for the purification of viruses, it is prone to gradient mixing, which can result in the variation of virus stock quality. Furthermore, ultracentrifugation is time consuming and labor intensive compared to chromatography-based purification methods. As an alternative, fast protein liquid chromatography (FPLC) has been used in purifying virus glycoproteins, RSV subunit vaccine preparations, and in purifying other enveloped viruses [16,17,18]. Since the initial publications on FPLC, chromatographic instrumentation has advanced, and the instruments are now small enough to fit inside a biosafety cabinet to preserve biosafety operating conditions. We therefore included RSV purified by FPLC in our analysis to compare with viral stocks purified by ultracentrifugation.

The most difficult cell-derived constituents to remove from virus stocks prepared by density gradient centrifugation methods are exosomes and microvesicles, which transport proteins and nucleic acids between cells, serving as intercellular communication vessels [19,20]. In particular, exosomes frequently co-fractionate with enveloped viruses during density gradient centrifugation due to similar biophysical properties, such as a 100–300 nm diameter and a lipid bilayer [19,20]. FPLC-based methods that isolate exosomes and microvesicles have been described; however, it is not clear if exosomes and microvesicles may co-fractionate with viruses during FPLC virus purification [21]. Therefore, we monitored the co-fractionation of exosome markers in the different stock preparations of RSV.

In this study, we compared the constituents, infectivity, the binding interaction of RSV to a receptor, nucleolin [22], and antibody neutralization responses of the RSV stocks purified by ultracentrifugation and FPLC methods. Knowledge of the purity and susceptibility of RSV stocks to experimental treatments will help to inform basic RSV research and the design of clinical trials of vaccines and therapeutics to treat RSV infections.

## 2. Materials and Methods

### 2.1. Antibodies

Anti-goat IgG H&L conjugated to β-Galactosidase; cat. No. ab136712 (Abcam Inc., Toronto, ON, Canada), Anti-superoxide dismutase 1; cat. No. sc-11407 (Santa Cruz Biotechnology Inc., Mississauga, ON, Canada), Anti-RSV 4 clone blend; cat. No. NB100-65217 and Anti-CD63 (H5C6); cat. No. NBP2-42225 (Novus Biologicals, Oakville, ON, Canada), Anti-RSV polyclonal; cat. No. B65860G (Meridian Life Science, Memphis, TN, USA), 6 nm Colloidal Gold-AffiniPure anti-mouse IgG (H&L); cat. No. 715-195-150, Horseradish peroxidase-conjugated light chain specific antibodies (Jackson ImmunoResearch Laboratories, West Grove, PA, USA), Alexa Fluor-488 and -647 conjugated secondary antibodies (Thermo Fisher Scientific, Waltham, MA, USA).

### 2.2. Cell Culture, Virus Propagation, and Respiratory Syncytial Virus (RSV) Reverse Genetics

HeLa cells were grown in Dulbecco’s Modified Eagle Medium (DMEM) with 10% heat-inactivated Fetal Bovine Serum (FBS). The 1HAEo- (human airway epithelial) cell line (gift from D. Gruenert, University of California, San Francisco, CA, USA) was grown in Minimum Essential Medium (MEM) with l-glutamine, high glucose, sodium bicarbonate, and 10% heat-inactivated FBS. All cell types were grown in a humidified incubator at 37 °C with 5% CO_2_. The laboratory adapted RSV type-A2 strain that was used has been described previously [23,24]. The molecular clone of RSV type-A2 expressing green fluorescent protein (GFP) (rgRSV RW30 [25]) (a gift from M.E. Peeples, Children’s Research Institute, Columbus, OH, USA) was rescued from full-length cDNA to infectious virus in HeLa cells. Briefly, full-length RW30 was transfected into sub-confluent HeLa cells along with support plasmids expressing RSV N, P, L, and M2-1. In addition, codon-optimized T7 RNA polymerase in pCAGGS (a gift from Benhur Lee, Addgene plasmid #65974) was co-transfected to drive plasmid expression in the place of vaccinia virus MVA-T7 [26]. After rescue, the RSV was propagated in T75 flasks of HeLa cells and harvested as cell-free (clarified) RSV-conditioned DMEM with 10% FBS before use in experiments as “Media-RSV” or further purification. RSV-conditioned media was snap frozen and stored in liquid nitrogen (LN_2_).

### 2.3. Purification of RSV by Sucrose Density Gradient Ultracentrifugation

RSV was precipitated from conditioned media by stirring with 10% Polyethylene glycol (PEG)-6000 for 90 min on ice, then pelleted by centrifugation at 4300× *g* at 4 **°**C for 30 min. The pellets were resuspended in NT Buffer (0.15 M NaCl, 0.05 M tris, pH 7.5) and overlaid on a discontinuous sucrose gradient (35%, 45%, 60% sucrose in NT Buffer), as described previously [15]. The sucrose purified RSV band was harvested after a 4 h spin at 217,290× *g* at 4 **°**C, then aliquoted and stored in LN_2_.

### 2.4. Purification of RSV by Iodixanol Density Gradient Purification

RSV was purified as per Gias et al. [14]. Briefly, we adjusted clarified conditioned media to 100 mM MgSO_4_ and 50 mM tris-HCl, pH 7.5, and then PEG-6000 precipitated the RSV as above. The PEG-precipitated RSV-pellets were resuspended with NT buffer containing 100 mM MgSO_4_ and layered onto a discontinuous Iodixanol/Opti-Prep (Cat. No. D1556, Millipore-Sigma, Oakville, ON, Canada) in TMSS (10 mM tris-HCl, 100 mM MgSO_4_, 0.25 M sucrose) gradients: 3 mL 52% iodixanol, 5 mL 36% iodixanol, and 3 mL 20% iodixanol. The gradients were centrifuged at 217,290× *g* for 2 h at 4 **°**C and the opaque band at the 20–36% interface was harvested.

### 2.5. Purification of RSV by Fast Protein Liquid Chromatography (FPLC)

The PEG-precipitated RSV resuspended in NT was filtered through an ÄKTA Start FPLC system (GE Healthcare Life Sciences, Mississauga, ON, Canada) stored within a biosafety cabinet. The instrument was equipped with a HiTrap Capto Core 700 column (Cat. No. 17-5481-51 GE Healthcare Life Sciences) with a 1 mL bed volume. The matrix beads of the column bind small impurities while allowing large molecules to pass through the column (MW > 700,000). ÄKTA Running Buffer A was comprised of NT buffer +1% sucrose, pH 7.4, 0.2 μm filtered, and ÄKTA Elution Buffer B was 1N sodium hydroxide in 30% isopropanol, 0.2 μm filtered. The system and column were washed with sterile filtered distilled water at 5 mL/min. The column was primed and equilibrated with 5 mL of Buffer A running at 1 mL/min. The 2-mL injection loop was flushed with buffer A and then loaded with the PEG-precipitated virus resuspended in NT buffer. Fractionation was set to collect 1 mL fractions as soon as the sample was injected onto the column at 0.5 mL/min, collecting 5 mL total throughout the absorbance peak at 250 nm. Isocratic elution was set at 100% Buffer B to remove the impurities bound to the column to allow for re-use. The column was stored at 4 **°**C in 20% ethanol.

### 2.6. Detection of RSV Infection

The RSV-infected cells were detected by the adaptation of a protocol that was described previously [27]. Confluent HeLa cells were inoculated with a 10-fold serial dilution of RSV-containing media for 3–4 h at 37 **°**C in a humidified incubator with 5% CO_2_. The cells were washed and incubated overnight in fresh growth media. After 16 to 20 h, the cells were fixed and permeabilized with 1 part methanol and 1 part acetone (*v*/*v*), blocked with phosphate-buffered saline (PBS) containing 5% FBS, and stained with goat polyclonal anti-RSV antibody. The cells were washed and probed with a secondary antibody conjugated to β-galactosidase. Infected cells stained blue by the addition of X-Gal substrate (5-bromo-4-chloro-3-indoyl-β-galactopyranoside) in PBS containing 3 mM potassium ferricyanide, 3 mM potassium ferrocyanide, and 1 mM magnesium chloride.

### 2.7. Viral RNA Quantification by q-RT-PCR

Media-RSV, sucrose-purified RSV, and FPLC-purified RSV stocks of known infectious titre were extracted and purified as per the QIAAmp Viral RNA Mini Kit (Qiagen, Toronto, ON, Canada). RNA was quantified using a Qubit fluorometer (Thermo Fisher Scientific, Waltham, MA, USA). Real-Time quantitative reverse transcription polymerase chain reaction (qRT-PCR) was performed using custom TaqMan primer-probes for RSV-A as described in [28] and qScript XLT 1-step qRT-PCR ToughMix (QuantaBio, Beverly, MA, USA). Standard 10-fold dilution curves were plotted for each preparation to verify PCR efficiency.

### 2.8. SDS-PAGE, Coomassie Staining, and Western Blot

Harvested aliquots of purified RSV were boiled for 5 min with Laemmli sample buffer and 2.5% 2-mercaptoethanol, then run on precast Mini-PROTEAN TGX polyacrylamide gradient gels (Biorad, Mississauga, ON, Canada) at 150 V for 1 h in tris-glycine-sodium dodecyl sulfate running buffer. Coomassie gels were stained with 3 g/L Brilliant Blue R-250, destained with 45% methanol, 45% water, and 10% acetic acid (*v*/*v*/*v*), then imaged. For the Western blot analysis, proteins were transferred onto a nitrocellulose membrane at 100 V for 1 h at 4 **°**C in tris-glycine transfer buffer including 10% methanol. The membranes were washed with 0.1% Tween (TBS-T), blocked with 5% bovine serum albumin (BSA) in TBS-T, and probed with primary then secondary-HRP antibodies in TBS-T containing 1% BSA. Protein bands were detected by ECL 2 substrate (Pierce PI80196), and imaged using a GE ImageQuant LAS 4000 (GE Healthcare Life Sciences).

### 2.9. Mass Spectrometry

All samples were reduced, denatured, and run into a Biorad TGX precast SDS-PAGE gel. The Coomassie-stained bands were then excised and in-gel trypsin digestion was performed. Briefly, the excised gel bands were destained twice in 100 mM ammonium bicarbonate/acetonitrile (50:50). Samples were reduced (10 mM β-mercaptoethanol in 100 mM bicarbonate) and alkylated (55 mM iodoacetamide in 100 mM bicarbonate). After dehydration, enough trypsin (6 ng/µL) was added to cover the gel pieces and the digestion proceeded overnight at room temperature. Tryptic peptides were first extracted from the gel using 97% water/2% acetonitrile/1% formic acid followed by a second extraction using 50% of the first extraction buffer and 50% acetonitrile. Fractions containing tryptic peptides dissolved in aqueous 25% *v*/*v* ACN and 1% *v*/*v* formic acid were resolved and ionized by using nanoflow HPLC (Easy-nLC II, Thermo Scientific) coupled to the LTQ XL-Orbitrap hybrid mass spectrometer (Thermo Scientific). Nanoflow chromatography and electrospray ionization were accomplished by using a New Objective PicoFrit fused silica capillary column (ProteoPepII, C18) with 100 μm inner diameter (300 Å, 5 μm). Peptide mixtures were injected onto the column at a flow rate of 3000 nL/min and resolved at 500 nL/min using 35 min linear gradients from 0 to 45% *v*/*v* aqueous ACN in 0.2% *v*/*v* formic acid. The mass spectrometer was operated in data-dependent acquisition mode, recording high-accuracy and high-resolution survey Orbitrap spectra using external mass calibration, with a resolution of 30,000 and an *m*/*z* range of 400–2000. The fourteen most intense multiply charged ions were sequentially fragmented by using collision-induced dissociation, and spectra of their fragments were recorded in the linear ion trap; after two fragmentations, all precursors selected for dissociation were dynamically excluded for 60 s. Data was processed using Proteome Discoverer 1.4 (Thermo Scientific) and a Uniprot human database was searched using SEQUEST (Thermo Scientific). The search parameters included a precursor mass tolerance of 10 ppm and a fragment mass tolerance of 0.8 Da. Peptides were searched with carbamidomethyl cysteine as a static modification and oxidized methionine, deamidated glutamine, and asparagine as dynamic modifications.

### 2.10. Transmission Electron Microscopy and Immuno-Negative Staining of RSV

PELCO Nickel/Carbon 200 (Electron Microscopy Sciences, Hatfield, PA, USA) mesh grids were plasma cleaned and negatively charged using a PELCO easiGlow (Ted Pella Inc., Redding, CA, USA) glow discharge system at 10 mA for 30 s. Each virus preparation was gently fixed with 0.5% paraformaldehyde (PFA) and permeabilized with 0.1% triton X-100. Staining was performed as previously published [29]. Samples were transferred to the grids for 8–12 min, incubated in 50 mM glycine in PBS for 5 min, blocked with 0.1% BSA in PBS for 10 min, stained with anti-RSV 4-clone blend antibody diluted 1:20 in blocking buffer for 15 min, washed 3 times in blocking buffer, stained with anti-mouse 6 nm colloidal gold conjugated antibody diluted 1:20 in blocking buffer for 10 min, and washed 2 times with blocking buffer and then 5 times in milli-Q H_2_O. Samples were negatively stained with 0.5% uranyl-acetate for 15 s and imaged using a Hitachi H-7650 Transmission Electron Microscope at a 60 kV accelerating voltage. Images were captured at 30,000–50,000× magnification with a 16-megapixel EMCCD camera in the low-mount position.

### 2.11. Blocking the Interaction of RSV with Nucleolin Using a DNA Aptamer

Guanine-rich oligonucleotide quadruplex DNA aptamer was used to block the interaction of RSV with cell surface nucleolin [30]. AS1411 (GGTGGTGGTGGTTGTGGTGGTGGTGG) and control aptamer CRO (CCTCCTCCTCCTTCTCCTCCTCCTCC) were ordered from Integrated DNA Technologies (Integrated DNA Technologies Coralville, Coralville, IA, USA) and incubated at 65 **°**C for 15 min before adding to confluent 1HAEo- cells. After a 1 h pre-incubation with 5 µM aptamer in normal growth media, the cells were infected with RSV for 20 h at 37 **°**C, 5% CO_2_, and then fixed and stained for RSV infection. Infected cells were manually counted as focus forming units, and Student’s *t* test determined the statistical significance between AS1411 and CRO treatments.

### 2.12. Imaging Flow Cytometry

1HAEo- cells were grown in MEM +10% FBS to ~90% confluence and treated with either 5 µM AS1411 or 5 µM CRO for 1 h at 37 **°**C. Before diluting in cellular growth media, AS1411 and CRO were heated to 65 **°**C for 15 min to induce aptamer formation. Infections were synchronized by cooling the cells for 10 min on ice, replacing the growth media with 4 **°**C media containing sucrose-purified RSV at a multiplicity of infection (MOI) of approximately 1 and AS1411 or CRO at 5 µM, and incubating for 1 h on ice. The RSV-containing media was then replaced with media pre-warmed to 37 **°**C containing AS1411 or CRO at 5 µM, and the cells were incubated for 60 min at 37 **°**C, 5% CO_2_, humidified. The cells were then washed with 4 **°**C PBS, and detached by incubation in 10 mM ethylenediaminetetraacetic acid (EDTA) in PBS on ice for 15 min, followed by scraping. The cells in suspension were blocked with 5% FBS in PBS for 30 min on ice, stained for surface nucleolin with rabbit polyclonal anti-C23 antibody, and stained for RSV using the mouse anti-RSV 4 monoclonal blend for 30 min on ice. The cells were then stained with a secondary donkey anti-rabbit Alexa 647 antibody and a secondary goat anti-mouse Alexa 488 antibody for 30 min on ice. The cells were then fixed in 4% paraformaldehyde and stained with 4′,6-diamidino-2-phenylindole (DAPI) for 5 min on ice followed by the acquisition of at least 100,000 events using the Amnis Mark II ImageStream (405 nm, 488 nm, and 642 nm excitation lasers, 60× magnification). Data analysis was performed using IDEAS software, version 6.2 (Amnis, Seattle, WA, USA).

### 2.13. Imaging Flow Cytometry Data Analysis

Data analysis occurred using the following gating strategy, with all gate values empirically pre-set for an unbiased analysis of the samples. Forward scatter (FSC) area vs FSC aspect ratio intensity was used to gate single cells from beads and doublets/clumps. An FSC gradient root mean squared (RMS) histogram was then used to gate in-focus single cells (high FSC gradient RMS). A DAPI (405 nm) aspect ratio histogram was used as a further quality control to remove cells with fragmented nuclei (high DAPI aspect ratio events are gated). Since the proportion of nucleolin on the cell surface is very low compared to cytoplasmic and nuclear regions, a nucleolin (642 nm) area histogram was used to gate non-permeable cells (low nucleolin area). RSV-positive cells were then gated using an RSV (488 nm) max pixel intensity histogram (low max pixel cells removed). The number of viral particles attached to each cell was then counted using a spot count, detecting bright spots with a 4:1 spot to background ratio, a minimum width of three pixels, a maximum area of 250 µm^2^, and a minimum intensity (similar to RSV-positive gate). Cell surface nucleolin was detected using a threshold 80% mask (removes the lower 20% of pixels) with a minimum intensity. Overlap between RSV and nucleolin was detected on cells with one to five viral particles bound, using the virus spot mask that had been dilated by two pixels, in conjunction with the cell surface nucleolin mask. Gate summary: Singlets, In Focus, Nuclear Shape, Non-Permeable, RSV Positive, Virus Spots.

### 2.14. Neutralization of RSV Infectivity

Goat polyclonal anti-RSV antibody at a concentration of 2 µg/mL was incubated at 37 **°**C, 5% CO_2_ with serial dilutions of each virus preparation in MEM containing 2% FBS for 1 h. The mixtures were then added to confluent 1HAEo- cells for 20 h at 37 **°**C, 5% CO_2_, and the cells were fixed and stained as per the β-galactosidase Assay. Infected cells were counted as focus forming units, and Student’s *t* tests determined the statistical significance between untreated and antibody-neutralized wells.

## 3. Results

**Figure 1:**
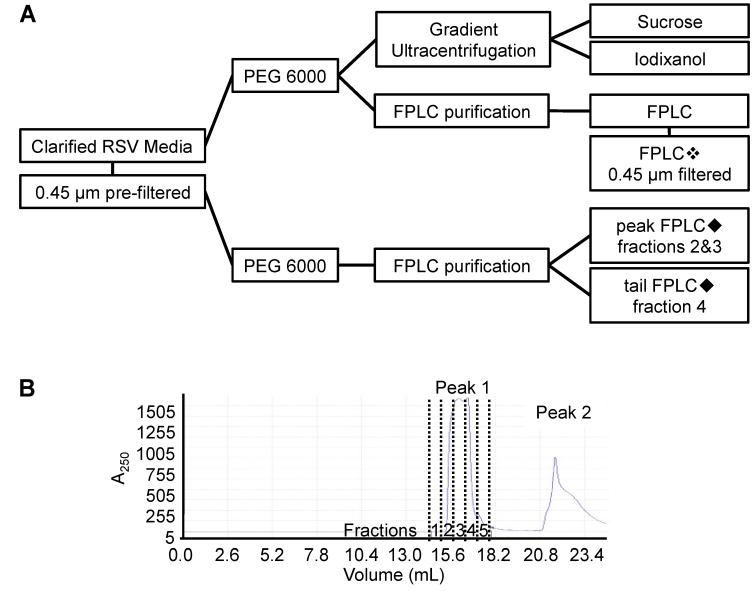
Respiratory Syncytial Virus (RSV) purification workflow. (**A**) Schematic of RSV purification methods. Pre- or post-fast protein liquid chromatography (FPLC) 0.45 µm syringe filtration steps are shown and denoted by a diamond symbol that is used to indicate these steps throughout; (**B**) Polyethylene glycol-6000 (PEG) precipitated RSV particles were purified by FPLC using a HiTrap Capto Core 700 column. The flow-through of virus occurred throughout the peak at 250 nm absorbance (peak 1) and was separated into five fractions. The column contaminants were eluted and visualized by the second absorbance peak (peak 2).

We conducted an analysis of RSV stocks that were derived from crude cell conditioned media or that were purified by density gradient ultracentrifugation or by FPLC. RSV was propagated in HeLa cells in preparation for the purification. The crude cell RSV-conditioned media was prepared the same way prior to its purification by both FPLC and ultracentrifugation to enable a comparison between the two methods. The RSV-containing media was first clarified by centrifugation and the infectious media was concentrated by PEG precipitation prior to its purification by ultracentrifugation or FPLC (Figure 1A).

### 3.1. Purification of RSV

The current standard for the purification of RSV (and most other enveloped viruses) is by sucrose gradient ultracentrifugation owing to the lower density of lipid membrane bound virions relative to denser proteinaceous debris [14,15,31,32]. Resuspended RSV-containing PEG precipitates underwent ultracentrifugation, as described above, through a discontinuous sucrose gradient, and a flocculent band containing RSV was collected from within the 45% sucrose fraction at the conclusion of ultracentrifugation. RSV-containing PEG precipitates also underwent ultracentrifugation through a discontinuous iodixanol gradient, followed by the harvest of an opaque band from within the 36% interface, as described above. Similar to what has been reported previously for other enveloped viruses [18], RSV was purified through an FPLC size-exclusion chromatography column. Resuspended RSV-containing PEG precipitates were loaded onto a HiTrap Capto Core 700 column mounted onto an ÄKTA Start FPLC. The column beads bound impurities smaller than 700 kDa, allowing RSV particles to pass though and be collected by an automated fractionator. A readout of the absorbance at 250 nm of each of the fractions is shown in Figure 1B. The first peak in ultraviolet (UV) absorbance corresponds to the flow-through of RSV from the column (Figure 1B). The second peak of UV absorbance corresponded to the buffer changeover and subsequent elution of contaminants bound to the column.

### 3.2. Determination of Infectivity of RSV Stocks

**Figure 2:**
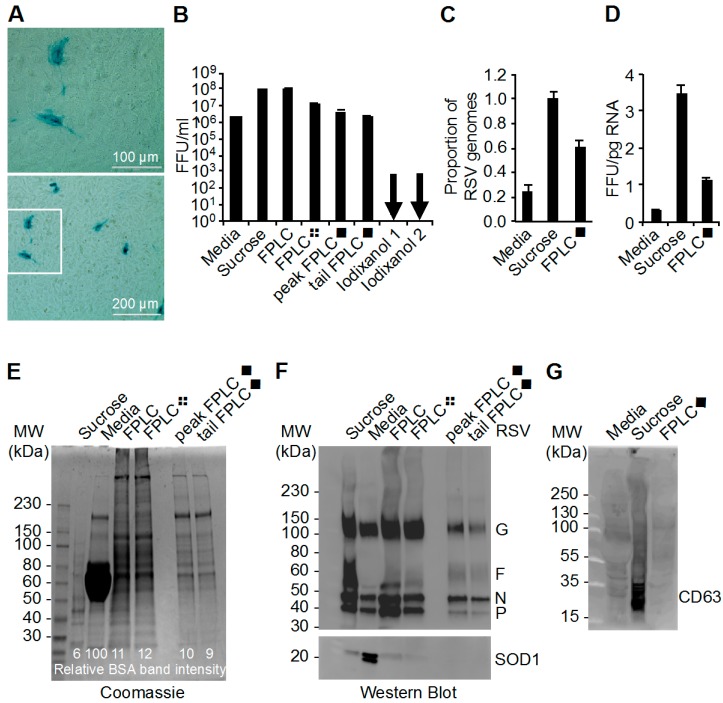
Infectivity and constituents of RSV stocks. (**A**) RSV was titrated on HeLa cells, fixed, and stained for RSV protein 20 h later. RSV infection was detected with a polyclonal anti-RSV antibody followed by a secondary antibody conjugated to β-galactosidase. The insoluble blue color is a product of X-Gal substrate cleavage by β-galactosidase and indicates RSV infection; (**B**) RSV focus-forming units (FFU blue spots) were manually counted and the titre of each virus preparation (outlined in Figure 1A) in FFU/mL is shown; (**C**,**D**) Viral RNA was extracted from RSV stock preparations and then measured by qRT-PCR; (**C**) Equivalent amounts of total RNA for each RSV preparation were probed and amplified. The proportion RSV genome detected is represented and compared to sucrose-purified RSV; (**D**) Viral titres for each of the RSV purification preparations were known and represented as FFU per picogram of total RNA; (**E**,**F**) RSV particles were harvested from HeLa cells in conditioned media and PEG-6000 precipitated for further purification as outlined in Figure 1A; (**E**) Coomassie blue staining of RSV stock preparations. The relative bovine serum albumin (BSA) band intensity was measured by densitometry of the 65 kDa band; (**F**) RSV proteins and superoxide dismutase 1 (SOD1) were detected by Western blot; (**G**) CD63 was detected by Western blot as an exosomal protein marker. MW: molecular weight.

The amount of infectious RSV that was recovered from each of the purification methods was determined. The infectivity of purified RSV stocks was quantified by titrating each RSV stock on a confluent monolayer of HeLa cells. The cells were fixed and stained for RSV antigen as per the β-galactosidase assay described in the methods section. The foci of RSV infection were stained blue, counted manually by bright field microscopy (Figure 2A), and reported as focus forming units per millilitre (FFU/mL) (Figure 2B).

We purified equivalent volumes of RSV-conditioned media starting material for each FPLC preparation and the iodixanol purifications. The sucrose-ultracentrifugation preparation was derived from 3 times more starting material. Equal titre was harvested in the sucrose and FPLC preparations, suggesting that FPLC affords higher infectious yields of RSV (Figure 2B). Infectious RSV has been recovered from an iodixanol gradient purification method reported previously [14]; however, we were unable to recover infectious virus using this method.

### 3.3. The Ratio of Genome Copies of RSV to Infectivity

The genomic copies of RSV in the different stocks were quantified using quantitative RT-PCR as previously [28] (Figure 2C,D). The number of RSV genomes can be used to quantify the number of viral particles and viral load in cell culture and patient samples, respectively [28]. In this study, the number of RSV genome copies was compared to the infectivity of the RSV stocks to obtain a measure of the genome copy to infectivity ratio. Genome copy number is a measure of viral particles in solution, although it is not precise because it does not differentiate between infectious particles and RSV RNA debris.

The number of viral genome copies per stock was higher in the sucrose ultracentrifugation-purified RSV compared to the FPLC◆-purified RSV stocks (Figure 2C). This difference in viral genome copies is reflected in the infectivity differences between the sucrose ultracentrifugation- and FPLC◆-purified stocks as shown in Figure 2B. However, when the infectivity of the stocks was compared to the total amount of RNA in the stocks, we noted that the sucrose ultracentrifugation-purified RSV contained more infectivity per picogram of RNA (Figure 2D).

### 3.4. Analysis of RSV Stocks by Coomassie Stain and Western Blot

The presence of RSV antigens in the unpurified and purified RSV stock preparations was analyzed by Western blot using a polyclonal anti-RSV antibody. A few highly abundant viral structural proteins (G, F, N, and P), identifiable by size, are indicated (Figure 2F). For comparison, the total protein content of each RSV stock was analyzed by Coomassie stain (Figure 2E). To begin directly assessing the purities of each RSV preparation, the viral stocks were analyzed by Western blot to detect cellular Cu-Zn Superoxide Dismutase 1 (SOD1) (Figure 2F). SOD1 is a relatively small soluble cytoplasmic protein that is readily secreted into the culture media by necrotic and apoptotic cells, which are plentiful during stock virus preparation [33,34]. Healthy cells also secrete the tetraspanin CD63 [35,36,37,38] along with SOD1 packaged in exosomes into the culture media [39,40]. The CD63 that was observed by Western blot, as a series of bands ranging from 20 to 45 kDa in size, has been described previously [35,36]. We observed less CD63 and SOD1 in the FPLC-prepared RSV stocks compared to the sucrose gradient-purified RSV (Figure 2F,G).

### 3.5. Mass Spectrometry Analysis of RSV Stocks

To complement the total protein analysis by Coomassie gel and SOD1 detection by Western blot, the constituents of each RSV preparation were analyzed by tandem mass spectrometry (MS) (Table S1, Supplemental spreadsheet 1). Bovine serum albumin was the most abundant contaminating constituent (Table S1, Supplemental spreadsheet 1). This is supported by the band in the Coomassie gel in Figure 2E that is brightest in the media virus lane at approximately 65 kDa. Table 1 compares the change in BSA quantification with each purification method as measured by MS or Coomassie densitometry. The sucrose gradient and FPLC◆ purifications reduced the intensity of the Coomassie band by about 94% and 90% by densitometry, respectively (Figure 2E). These values are consistent with the reduction in albumin peptide spectral matches (PSMs) reported by MS, that were reduced by 88% and 84% in the sucrose gradient and FPLC◆ preparations, respectively (Table S2, Supplemental spreadsheet 1) [41]. The sensitivity of MS protein identification is relative. In the RSV-conditioned media sample, for instance, BSA peptides were so numerous that the RSV nucleoprotein peptides were undetectable, as they were below the threshold of relative abundance. Although both ultracentrifugation and FPLC decreased the presence of bovine protein contamination, neither purification method completely removed all traces of cell-derived contaminants.

Tandem mass spectrometry analysis identified host-cell derived glyceraldehyde 3-phosphate dehydrogenase (GAPDH) as a contaminant of sucrose gradient purifications that was not detected in the FPLC preparations (Table 2). GAPDH is another host cell protein that is found in exosomes [42]. Due to the biophysical similarities of RSV virions and exosomes, the identification of exosomal proteins in sucrose gradient preparations of RSV is not surprising. The pattern of cell-derived GAPDH contamination that was detected by MS (Table 2) correlates with the pattern of SOD1 and CD63 contamination observed by Western blot (Figure 2F,G), in that both were reduced to nearly undetectable levels by FPLC purification. However, we did not detect SOD1 by MS, which is likely because Western blot is a more sensitive technique for identifying specific proteins of relatively low abundance [41].

### 3.6. Effect of 0.45 µm Filtration Steps in RSV Stock Purification

Strategically placed 0.45 µm filtration steps were employed to remove contaminants larger than RSV viral particles (Figure 1A) [13]. Filtration after FPLC purification (FPLC❖) resulted in a decrease in SOD-1 contamination by Western Blot (Figure 2F) as well as a log decrease in infectivity (Figure 2B). Filtration prior to PEG precipitation (FPLC◆) resulted in the greatest reduction of host-cell derived SOD-1 protein by Western blot; however, it also markedly reduced the viral protein (Figure 2F) and infectious titre recovered (Figure 2B). Infectious RSV is lost with 0.45 µm filtration steps at any point in the purification process.

### 3.7. Infection by Purified RSV Was Significantly More Susceptible to Receptor Blocking When Compared to Unpurified RSV

The FFU to RSV genome copy and RNA ratios varied significantly depending on the method of RSV purification (Figure 2C,D). It was therefore possible that differences in virus stock contents in the inoculum could affect cellular responses to the virus even before virus entry [43,44]. Each purification method requires a certain amount of manipulation, including a change to the sucrose-containing storage buffer. Both of these variables could alter the virions, themselves, to differing degrees. The binding of a virus with its cellular entry receptor is one of the first interactions that a virus has with the host cell, and it is a requirement of productive infection. Numerous receptors for RSV have been described [1], and receptor usage may have been altered by the mode of purification. Cell surface nucleolin serves as an entry receptor for RSV on bronchial epithelial cells [22,45], so we tested the susceptibility of raw unpurified and purified RSV to nucleolin blocking during viral entry. To study this interaction, a guanosine-rich nucleolin-binding DNA aptamer (AS1411) was used to block the interaction of RSV with nucleolin [30,46,47,48,49]. A cytosine-rich aptamer that does not bind nucleolin (CRO) was used as a control.

**Figure 3:**
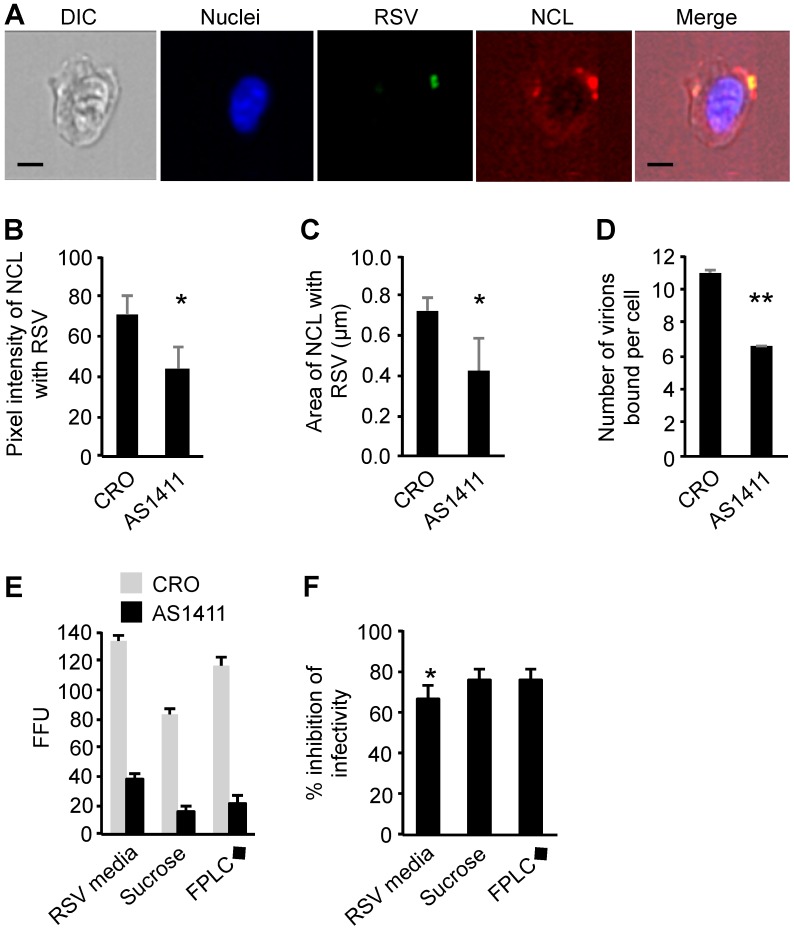
The susceptibility of RSV stocks to nucleolin (NCL) receptor blocking by the DNA aptamer, AS1411. (**A**–**F**) 1HAEo- bronchial epithelial cells were treated with 5 μM CRO control aptamer or AS1411 aptamer for 1 h and then inoculated with equivalent infectious units of RSV. (**A**) The interaction of RSV with NCL was observed and quantified by imaging flow cytometry 60 min after infection. A representative ImageStream array is shown that demonstrates the typical patching of NCL that was associated with RSV binding to the cell surface. The differential interference contrast (DIC) and the fluorescent images are provided and the scale bar represents 10 μm. When images were gated on RSV particles bound to the surfaces of cells, there was a significant decrease in the (**B**) mean NCL pixel intensity, the (**C**) mean area of the NCL patch; and the (**D**) mean number of RSV particles bound per cell. * *p* < 0.05, ** *p* < 0.01 by Student’s *t* test. Experiments are representative of two independent experiments that used a prototypic RSV-A2 and a clinical isolate of RSV type-A; (**E**) Aptamer pre-treatment and equalized RSV infection was allowed to proceed for 20 hours before the monolayers were stained and enumerated; (**F**) Percent inhibition of RSV infection was determined by dividing the AS1411 infectivity by the CRO infectivity in panel (**E**) then multiplying by 100. * *p* < 0.05 by Student’s *t* test.

1HAEo- bronchial epithelial cells were pretreated with AS1411 and then inoculated with RSV. The interaction of RSV with nucleolin 60 min after inoculation was quantified by imaging flow cytometry (Figure 3A), and the patching of nucleolin with RSV was an indication of RSV binding [22]. The AS1411 aptamer treatment caused a significant decrease in the pixel intensity of the nucleolin that was patching with RSV when compared to the CRO control aptamer treated cells (Figure 3B). The AS1411 aptamer also reduced the patch area (Figure 3C) and the number of RSV particles bound to 1HAEo- cells (Figure 3D) when compared to the CRO control aptamer treatment.

The reduction of nucleolin binding to RSV as a result of the pretreatment of cells with the AS1411 aptamer suggested that AS1411 should also inhibit RSV infection. 1HAEo- cells were pretreated with AS1411 or CRO, then inoculated with RSV derived from conditioned media, sucrose purification, and FPLC◆ purification. The purified stocks were significantly more susceptible to receptor blocking compared to the raw unpurified media virus (Figure 3F). However, there was no difference in blocking susceptibility between the sucrose and FPLC◆-purified RSV stocks (Figure 3E). These results suggested that ultrastructural differences might exist in the purified RSV preparations as compared with the unpurified RSV.

### 3.8. Transmission Electron Microscopy of RSV Stocks

**Figure 4:**
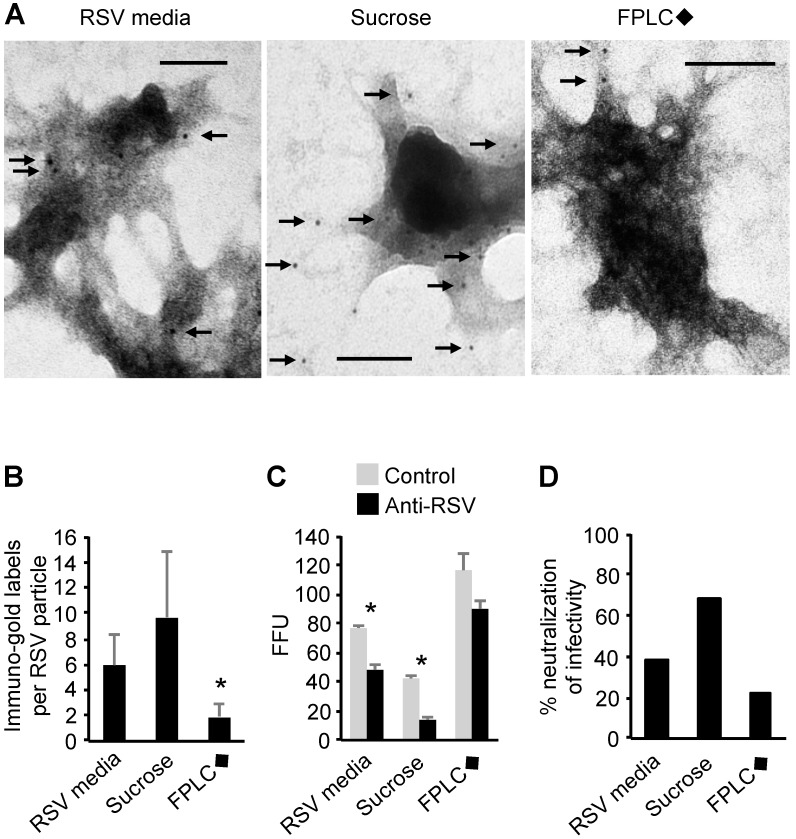
Imaging RSV stocks by transmission electron microscopy and measuring their susceptibility to antibody neutralization. (**A**) Unpurified virus (RSV media) and sucrose- and FPLC◆-purified RSV stocks were immuno-labeled, negatively stained, and then imaged by transmission electron microscopy. A representative image of each is shown with black arrows indicating anti-RSV immuno-gold particles. The scale bar is 100 nm long; (**B**) The immuno-gold dots bound to twenty-five virus particles from the transmission electron microscopy imaging of each purification method were counted. * *p* < 0.05 by Tukey’s test of significance; (**C**,**D**) Equivalent infectious units of unpurified virus (RSV media), sucrose ultracentrifugation-purified, and FPLC◆-purified stocks were incubated with 2 μg/mL anti-RSV polyclonal antibody and then added to HeLa cells for 20 h before being fixed and stained for RSV infection; (**C**) Focus forming units were manually counted and are represented as FFU per well. * *p* < 0.05 by Student’s *t* test. The neutralization as a percentage of control FFU/mL of each preparation is shown in panel (**D**).

Transmission electron microscopy was used to image RSV virions before and after sucrose gradient or FPLC◆ purification. Purified virus particles were gently fixed, permeabilized, stained, and imaged directly to obtain an ultrastructural image of the whole virion, as opposed to embedding and sectioning virus particles prior to imaging. In the electron micrographs, we identified particles that were reminiscent of RSV virions (Figure 4A), as described previously [13,50,51]: the particles that were observed ranged in size from 100 nm to 300 nm in diameter. The shapes of the virion-like particles ranged from oval and oblong to more amorphous and asymmetrical shapes [51]. The most striking feature was that most of the particles contained an electron dense core enclosed in a membrane-like structure. We also noted that in the sucrose preparations, there were particles that did not contain electron dense cores. These particles were reminiscent of exosomes and microvesicles that ranged in size between 50 and 300 nm in diameter [52,53].

The images of RSV-like particles differed between RSV preparations. In particular, we noted that there was an extensive web of debris surrounding electron dense particles of approximately 100 nm in diameter in the FPLC◆-purified RSV preparations that was not as dense as the debris surrounding the ultracentrifugation-purified RSV stocks. Fewer anti-RSV immuno-gold antibodies bound to FPLC◆-purified virions compared to unpurified or ultracentrifugation-purified RSV as imaged by TEM (Figure 4B). These results suggested that antigen exposure on the surface of purified RSV may vary between purification preparations. Therefore, we targeted the exterior of the virus particles by antibody neutralization.

### 3.9. Susceptibility of Purified and Unpurified RSV Stocks to Antibody Neutralization

To test this, serial dilutions of virus from each of the purification methods were incubated with an anti-RSV polyclonal antibody, or control antibody, and used to inoculate HeLa cells. After one day of incubation, the cells were fixed and stained for RSV infection. There was a trend toward neutralization (20% reduction of infectivity (*p* = 0.30)) of the FPLC◆-purified RSV (Figure 4C). However, significant neutralization by antibody co-incubation occurred in ultracentrifugation-purified RSV (69% (*p* = 0.02)) and in unpurified RSV media stocks (38% (*p* = 0.03)) (Figure 4D). These results suggested that the FPLC◆-purified RSV particles were significantly less susceptible to antibody neutralization compared to ultracentrifugation-purified RSV and unpurified RSV in media.

## 4. Discussion

We compared the constituents and biological activity of RSV that was purified by ultracentrifugation or FPLC. There was a carapace of detritus surrounding FPLC◆-purified virions that was not present on ultracentrifugation-purified RSV virions. The debris surrounding the FPLC◆-purified virions likely led to a loss of susceptibility to neutralizing antibodies. Unpurified RSV and RSV purified by ultracentrifugation were significantly more susceptible to antibody neutralization, however. As expected, the RSV stocks purified by different methods were similarly susceptible to nucleolin receptor blocking and more susceptible than raw unpurified RSV. The ultracentrifugation-purified RSV stock was cleaner with less extraneous RNA and more efficient in terms of infectivity when compared to unpurified or FPLC◆-purified RSV.

The different susceptibilities of RSV stock preparations to neutralizing antibody treatment were unexpected. This is in light of there being little apparent difference in infectivity between sucrose gradient ultracentrifugation-prepared RSV and the RSV that was prepared by FPLC◆. This suggests that, in RSV stocks of both purification methods, RSV-F is available on the viral membrane to interact with nucleolin and mediate fusion of the viral envelope with the host cell membrane. Therefore, we had no reason to predict that the different RSV stocks would respond differently to neutralizing antibodies.

We speculate that the debris matrix of material surrounding virion-like FPLC◆-purified particles may play a role in the differential neutralizing antibody response. Although the debris matrix likely did not affect the viral receptor-nucleolin interaction and the entry of FPLC◆-purified RSV into the host cell, it may have interrupted the access of antibodies to key neutralizing epitopes on the virions. This could be due to differences in the affinities of the nucleolin receptor binding compared to the neutralizing antibody affinity for viral epitopes [54]. To conclude, the debris matrix surrounding the FPLC virions may preclude access to some of the larger IgM antibodies in the polyclonal anti-RSV antibody mix, whereas the virus-receptor interactions may be of smaller or higher affinity and thus not interrupted by the debris matrix.

A comparison of the chromatographic and ultracentrifugation purification methods of a retrovirus has been done previously [32]. McGrath et al. reported that sepharose column filtration was superior in preserving the glycoprotein integrity of Moloney Sarcoma Virus (MSV) [32]. Chromatography instruments have become considerably more automated and user friendly in the 38 years since McGrath et al. reported on the purification of MSV [32]. Here, we conducted a similar comparison of RSV purification methods. Additionally, we provided a detailed description of stock constituents that were detected by MS, a qualitative analysis of the RSV stocks by electron microscopy, and the differential responses of the RSV preparations to receptor blocking and virus neutralizing antibodies. We conclude that the suitability of the method of purification depends on the end use of the virus. This is because although sucrose ultracentrifugation produced RSV with more infectivity per total RNA, there was more exosome contamination, whereas exosome contamination could not be detected in the stocks of RSV purified by FPLC◆. There was more albumin contamination in the FPLC stocks, but we argue that this may be improved by reducing albumin in the growth media of the cells that are used to propagate RSV.

Previously, Radhakrishnan et al. published an extensive list of non-viral proteins in their proteomic analysis of sucrose-purified RSV preparations [9]. However, bovine contaminants were not reported because only human proteins were queried in their MS analysis [9]. Therefore, to the best of our knowledge, our study is the first to report in an unbiased manner all proteins present in RSV preparations by mass spectrometry.

Sucrose-containing buffer is beneficial for the cryopreservation of RSV [12]. However, the purification of RSV by sucrose gradient ultracentrifugation results in an RSV stock containing a concentration of sucrose much higher than is required for RSV stability. High sucrose concentrations can have undesirable effects in cells in tissue culture because sucrose concentrations of 0.45 M inhibit endocytosis [27]. Practically speaking, when inoculating cells in tissue culture with virus in sucrose, one must balance RSV titre with sucrose concentration. To remove sucrose from the inoculum and increase the virus titre, RSV can be pelleted by ultracentrifugation after purification, and then re-suspended in PBS [55]. In our hands, this practice damaged the integrity of the enveloped viruses and caused particle fragmentation (data not shown). FPLC simplifies the purification process by removing the need for ultracentrifugation while providing control over the sucrose concentration of the purified product.

The filtration of unpurified RSV medium through a 0.45 µm filter before purification provided a cleaner product (FPLC◆). Although the pre-filter process resulted in a 10-fold drop in infectivity, theoretically, this could be offset by loading a larger amount of starting material to the PEG precipitation step. The HiTrap Capto Core 700 column is efficient at removing low molecular weight contaminants, and this filtration step complements the FPLC method by removing very large impurities as well.

The neutralization resistance of FPLC◆-prepared virus may have downstream clinical utility. With engineered viral vectors, it is preferable to avoid antibody neutralization to increase the probability that the vector will transduce the target cell. If the same phenomenon occurs with other membrane-bound viruses, the lack of neutralization susceptibility of FPLC-purified virus may make it desirable as an immune evasion tool for therapeutic viral vectors. In summary, our study suggests that one must exercise caution when selecting a viral purification method. As we demonstrate here, the method itself may alter key study results, such as viral ultrastructure and susceptibility to neutralizing antibodies.

## Figures and Tables

**Table 1:** Percent reduction in bovine serum albumin detected in purified RSV preparations compared to relative amount in RSV-conditioned media.

Method	Sucrose-Purified RSV	RSV-Conditioned Media	FPLC	FPLC❖	Peak FPLC◆	Tail FPLC◆
**LC MS/MS**	88%	-	70%	84%	84%	85%
**Coomassie**	94%	-	89%	88%	90%	91%

**Table 2:** Mass spectrometry summary of RSV purification methods

Origin	Protein Name (Uniprot)	Sucrose-Purified RSV		RSV-Conditioned Media		FPLC		FPLC❖		Peak FPLC◆		Tail FPLC◆	
		PSM	CVG	PSM	CVG	PSM	CVG	PSM	CVG	PSM	CVG	PSM	CVG
Bovine	Serum Albumin	13	22%	349	68%	69	45%	33	40%	32	39%	34	44%
RSV	Nucleo-protein	18	27%	ND	ND	15	32%	7	25%	2	8%	2	7%
Human	Histone H4	5	41%	ND	ND	2	21%	2	21%	ND	ND	2	21%
Human	GAPDH	4	18%	ND	ND	ND	ND	ND	ND	ND	ND	ND	ND

PSM = peptide spectral matches; CVG = % coverage of the observed proteins; ND = not detected.

## Supplementary Materials

The following are available online at www.mdpi.com/1999-4915/9/8/207/s1, Supplemental Spreadsheets S1–S6: LC-MS/MS analysis of RSV purification preparations.
